# The Impact of Climate Change on Mental Health: A General Population Study

**DOI:** 10.1192/j.eurpsy.2024.1415

**Published:** 2024-08-27

**Authors:** R. Uçak, C. H. Ayhan, M. C. Aktaş, N. Kaynar Demirel

**Affiliations:** ^1^psychiatric nursing, Van Yuzuncu yıl university, van; ^2^Psychiatric nursing, Van Yuzuncu yıl university, -, Türkiye

## Abstract

**Introduction:**

Climate change and its impact on mental health is a growing area of research. Several studies have explored the relationship between climate change and mental health, highlighting the various ways in which climate change can affect individuals’ psychological well-being. Incorporating mental health indicators into climate change and health vulnerability and adaptation assessments is another important aspect of research in this area (Hayes & Poland, 2018). The study suggests that standardized methods to measure and predict the psychosocial outcomes of climate change should be implemented to better understand the mental health impacts. While the physical health consequences of climate change have received more attention, the mental health impacts are often overlooked (Nicholas et al., 2020).

**Objectives:**

This study was planned to examine the impact of climate change the impact of climate change on mental health

**Methods:**

This descriptive and cross-sectional study was conducted with individuals who willing to participate the study and above 18 years age. Individuals who saw the online advertisement and click on the study’s link were be brought to the study’s home page on Online Surveys. Should they wish to proceed, they will be brought to an information page detailing the purpose of the study, how their confidentiality and anonymity will be preserved and how their data will be treated.

Socio-Demographic Data Form, Climate Change Worry Scale, Eysenck Personality Questionnaire Revised- Abbreviated, general health questioner and Depression, Anxiety, Stress scale were used for collecting data. Data analyses was planned to run via Statistical Package for the Social Sciences version, 27.0.

**Results:**

The analysis of the data is still ongoing in detail by the researchers. The findings and relational implications of the study will be presented.

**Conclusions:**

In conclusion, this study highlight the importance of understanding the mental health impacts of climate change and developing strategies to address them. Climate change can have direct and indirect consequences on mental health, and vulnerable populations such as children and adolescents may be particularly at risk. Reducing psychological distance and incorporating mental health indicators into assessments can help in understanding and addressing the mental health consequences of climate change.

**Disclosure of Interest:**

None Declared

